# *De Novo* Assembly of the Complete Genome of an Enhanced Electricity-Producing Variant of *Geobacter sulfurreducens* Using Only Short Reads

**DOI:** 10.1371/journal.pone.0010922

**Published:** 2010-06-08

**Authors:** Harish Nagarajan, Jessica E. Butler, Anna Klimes, Yu Qiu, Karsten Zengler, Joy Ward, Nelson D. Young, Barbara A. Methé, Bernhard Ø. Palsson, Derek R. Lovley, Christian L. Barrett

**Affiliations:** 1 Bioinformatics and Systems Biology Graduate Program, University of California San Diego, La Jolla, California, United States of America; 2 Department of Bioengineering, University of California San Diego, La Jolla, California, United States of America; 3 Department of Microbiology, University of Massachusetts, Amherst, Massachusetts, United States of America; 4 Department of Microbial and Environmental Genomics, J. Craig Venter Institute, Rockville, Maryland, United States of America; University of Hyderabad, India

## Abstract

State-of-the-art DNA sequencing technologies are transforming the life sciences due to their ability to generate nucleotide sequence information with a speed and quantity that is unapproachable with traditional Sanger sequencing. Genome sequencing is a principal application of this technology, where the ultimate goal is the full and complete sequence of the organism of interest. Due to the nature of the raw data produced by these technologies, a full genomic sequence attained without the aid of Sanger sequencing has yet to be demonstrated.

We have successfully developed a four-phase strategy for using only next-generation sequencing technologies (Illumina and 454) to assemble a complete microbial genome *de novo*. We applied this approach to completely assemble the 3.7 Mb genome of a rare *Geobacter* variant (KN400) that is capable of unprecedented current production at an electrode. Two key components of our strategy enabled us to achieve this result. First, we integrated the two data types early in the process to maximally leverage their complementary characteristics. And second, we used the output of different short read assembly programs in such a way so as to leverage the complementary nature of their different underlying algorithms or of their different implementations of the same underlying algorithm.

The significance of our result is that it demonstrates a general approach for maximizing the efficiency and success of genome assembly projects as new sequencing technologies and new assembly algorithms are introduced. The general approach is a meta strategy, wherein sequencing data are integrated as early as possible and in particular ways and wherein multiple assembly algorithms are judiciously applied such that the deficiencies in one are complemented by another.

## Introduction

The sequencing of the first bacterial genome in 1995 has left a lasting impact on the field of prokaryotic genomics. The next revolution in the field of genomics has been the development and progress of high-throughput sequencing technologies. These next-generation sequencers, mainly those from Illumina and 454 Life Sciences (454), generate millions of short reads that are more error-prone than the traditional Sanger sequencing. However, these technologies have greatly reduced the cost of sequencing per base and thus have opened up a wide range of applications. The major applications include resequencing of closely related individuals for personalized genomics and *de novo* sequencing of new microbial genomes.

*De novo* sequencing using next-generation technologies has necessitated the development of new algorithms for assembling the short and more error-prone reads that they generate. Several *de novo* assembly algorithms based on de-Bruijn graphs (EULER-SR [1] and Velvet [2]), hash-extension (VCAKE) [3], overlap layout (EDENA) [4] and for paired-end reads (ALLPATHS) [5] have been recently developed. These algorithms are capable of assembling millions of short-reads from next-generation sequencing technologies into thousands of contigs with varying degrees of efficiency.

While 454 reads are longer than Illumina reads (∼250–450 bp compared to ∼36–100 bp), they have a higher indel error rate when compared to Illumina reads. The longer 454 reads, though, inherently offer advantages over the shorter Illumina reads for *de novo* assembly. Illumina reads, despite being much shorter, provide a higher depth of coverage than 454 reads. This complementary nature of Illumina and 454 reads has been exploited by some recent methods that have produced an assembly of *P. syringae pathovar oryzae*, consisting of 126 scaffolds, 2002 unincorporated contigs, and an N50 of 91.5 kb [6]. Another report integrated these two data types using a different approach to assemble an *Acinetobacter baylyi* strain into 10 scaffolds with an N50 of 1Mb [7]. Salzberg and colleagues assembled a virulent strain of *P. aeruginosa* PA01 into a large 6.3 Mb scaffold [8] using a mixed comparative and *de novo* approach that included a gene-boosted strategy. However, this approach heavily relied on comparative information and thus cannot be classified as *de novo*.

Despite these recent reports that indicate significant progress by integrating Illumina and 454 technologies, complete *de novo* assembly of microbial genomes from only short reads and without aid from Sanger sequencing still remains an unsolved challenge. This challenge is critically important [9], for a single, circular nucleotide sequence of the complete chromosome is a necessary prerequisite for confident and complete research based on a genome. As an answer to this challenge, we have developed a strategy (Meta-Assembly) for complete, whole-genome *de novo* assembly and applied it to a novel *Geobacter* variant (KN400) that is capable of unprecedented current production at an electrode [10]. Our Meta-Assembly strategy adopts a bi-level integrative approach that leverages the different and complementary results provided by multiple assembly programs over and above the integration of complementary data types to obtain a complete, whole-genome assembly. We have applied this strategy using 50× Illumina GA1 singleton reads and 16× 454 GS-FLX paired-end sequencing reads and assessed our finished assembly by sequencing nearly 1% of the genome by Sanger sequencing and by a comparison to the genome of a highly related strain.

## Results

### Meta-Assembly strategy results in the complete genome of KN400 from a mixture of short reads

**Figure 1:**
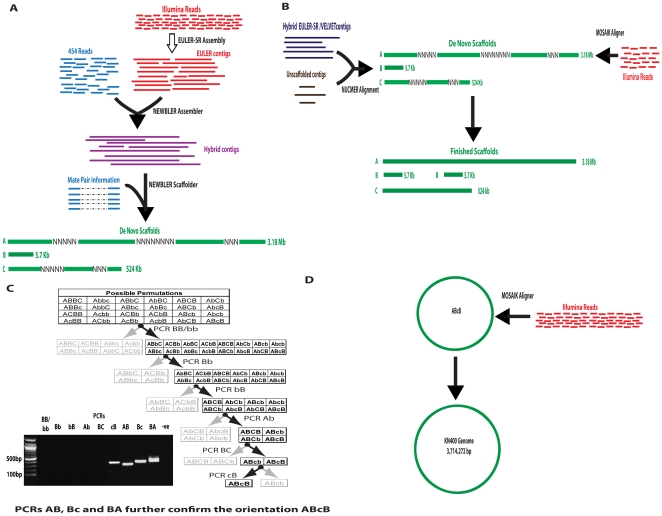
Assembly Strategy. A) Hybrid Assembly Phase; B) Scaffold Bridging and Finishing Phase; C) Scaffold Ordering Phase: The left branch of the decision tree consists of permutations that can be confirmed by the PCR performed while the right branch consists of those permutations that cannot be confirmed by the particular PCR. The faded permutations are those which have been eliminated by the PCRs while those in bold are those that are remaining. (Gel Inset: Showing PCR products for all the 9 PCRs performed in the search strategy to confirm the correct orientation of the scaffolds); D) Genome Finishing Phase.

Our Meta-Assembly approach (Figure 1) consists of four distinct phases: Hybrid Assembly, Scaffold Bridging and Finishing, Scaffold Ordering and Genome Finishing.

In the Hybrid Assembly phase (Figure 1A), we first filtered and assembled the Illumina reads alone using the de-Bruijn graph based algorithm EULER-SR [1]. This assembly consisted of 4233 contigs with an N50 of 1.48 Kb. We then assembled these contigs, along with all of the reads not assembled by EULER-SR [1] and all of the 454 reads (i.e. neglecting the pair information) using Roche's algorithm Newbler (http://www.454.com/products-solutions/analysis-tools/gs-de-novo-assembler.asp). This combining step was a critical aspect in maximizing the complementary information in Illumina and 454 reads, as shown by the resulting assembly of 270 hybrid contigs with an N50 of 92.67 kb (Table 1). We then leveraged the mate pair information by combining these 270 “hybrid” contigs with the paired 454 reads using Newbler's scaffolder. The contigs that did not form part of one of the output scaffolds (unscaffolded contigs) were utilized later in the final Finishing phase. This scaffolding step resulted in a greatly improved assembly, giving three *de novo* scaffolds of lengths 3.18 Mb, 5.7 kb and 524 kb (respectively scaffolds A, B, and C in Figure 1a.) with a total length of 3.7 Mb.

**Table 1:** Summary and statistics of different stages of Meta-Assembly.

Phase	Assembler	Number of Contigs/Scaffolds	N50(kb)	Degenerate Positions	Assembly Length(Mb)
A	EULER-SR(Illumina Alone)	4233	1.487	0	3.51
A	Newbler(454 Reads+Illumina)	270	92.67	0	3.72
A	Newbler Scaffolder (Mate Pairs)	3	3184.3	41421	3.71
B	Scaffold Bridger/Finisher	4	3184.3	0	3.71
C	Scaffold Ordering	1	3714.2	0	3.71
D	Finisher	1	3714.2	0	3.71

#### Exploiting the complementary nature of assembly algorithms significantly improves the quality of the *de novo* scaffolds

The *de novo* scaffolds A and C from the Hybrid Assembly phase contained numerous stretches of degenerate nucleotides, and to resolve them we applied a post-processing step that exploited the coverage provided by short-reads. We developed a Scaffold Bridging and Finishing phase for the purpose of linking the *de novo* scaffolds and for resolving the intra-scaffold degenerate nucleotide positions that were introduced by the scaffolder (Figure 1B). In this phase, we leveraged the complementary nature of the assemblies generated by programs like EULER-SR [1], Velvet [2] and Newbler. Since EULER-SR [1] and Newbler generate slightly different sets of contigs, we created a second set of hybrid contigs from Illumina and 454 reads using EULER-SR. We aligned these hybrid contigs against the *de novo* scaffolds using NUCMER and analyzed the alignment for the threes scenarios that could potentially bridge the scaffolds and resolve the degenerate nucleotides (Methods and Fig S2). We found that none of the hybrid EULER-SR [1] contigs aligned in such a way that they bridged any pair of *de novo* scaffolds (Fig. S2A). We were able to resolve all intra-scaffold degenerate nucleotide positions by either substituting the corresponding bases from hybrid EULER-SR [1] contigs that overlapped with flanking regions of Ns in the *de novo* scaffolds (Fig S2B), or by removing the degenerate bases when the regions flanking them aligned to contiguous regions on the hybrid EULER-SR contigs (Fig S2C). (We implemented the same approach using hybrid contigs generated by Velvet [2] as the complementary set instead of EULER-SR [1] and obtained a similar result.) At this stage, our assembly could contain small indels due to 454 sequencing or due to our custom program. To correct these, we aligned the Illumina reads using the Smith-Waterman capabilities of MosaikAligner (Stromberg and Marth in preparation). We also analyzed these scaffolds for potential repeats/duplications by examining the read coverage and also the multiplicity of the vertices in the repeat graph that is part of EULER-SR's output. This analysis revealed that scaffold B was indeed duplicated and a BLAST [11] search identified it as an rRNA gene. At the end of this phase, then, our assembly consisted of four scaffolds, identified in Figure 1B as A (3.15 Mb), B (5.7 Kb) occurring twice, and C (518 Kb).

#### An efficient PCR-based search strategy results in the correct orientation of the scaffold and a circular genome

In the Scaffold Ordering phase, we considered the KN400 genome to be a signed circular permutation of the four scaffolds–giving 24 unique possible permutations (Figure 1C). To determine the correct relative orientation of the scaffolds, we employed a polymerase chain reaction (PCR)-based search strategy. Our approach consisted of nine PCRs, six of which serially eliminated 23 possible permutations. The orientation ABcB was confirmed by the remaining three PCRs (Figure 1C). This PCR-based ordering approach enabled us to link the four scaffolds into one circular chromosome of 3.71 Mb. This approach must not be confused with the standard gap-closing approaches adopted, because we do not use PCRs to fill gaps but only to confirm the relative orientation of the scaffolds and just link them up. That is, there were no intervening nucleotides between the four scaffolds. In fact, our approach goes a step further in validating the sequence by accounting for the reverse complement of the scaffolds, thereby eliminating any potential false rearrangements that might be introduced in the assembly.

#### Depth of coverage offered by Illumina reads corrects the indels introduced by 454 and scaffold finishing and ordering

We corrected for indels and any errors introduced during our scaffold finishing and scaffold ordering phase by aligning the Illumina reads to the ordered scaffold ABcB using MosaikAligner (Stromberg and Marth in preparation). The result was a complete circular genome consisting of 3,714,272 bp. The statistics of changes made by this alignment are provided in Table 2. The genome can be accessed from GenBank under the accession number CP002031.

**Table 2:** Changes made due to alignment of Illumina reads in the genome-finishing phase.

Changes	Number of Changes
SNPs	101
Deletions	18
Insertions	7

### Sanger sequencing of selected regions of KN400 genome validates the *de novo* assembly

**Figure 2:**
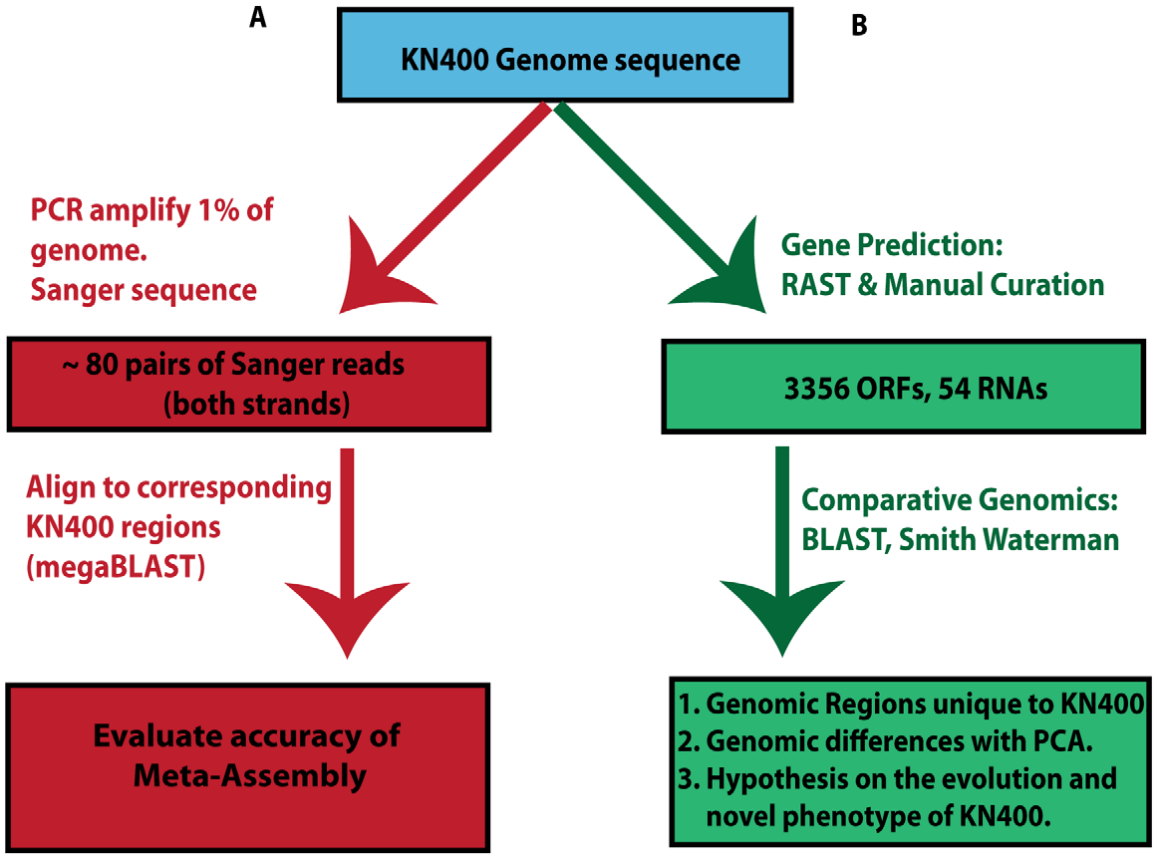
Assembly validation approaches. A) Sanger sequencing approach; B) Comparative Genomics approach.

To validate the *de novo* assembly approach adopted here and to estimate the accuracy of the obtained genome sequence, we amplified 32kb (∼1% of the genome) of KN400 and performed Sanger sequencing on both the forward and reverse strands (Figure 2A). We compared these sequenced regions to the corresponding genomic region obtained from Meta-Assembly using megaBLAST [12] alignment algorithm.We found that out of the 32680 bp sequenced by Sanger sequencing, there were 32675 perfect matches, four SNPs and one 1 bp insertion with respect to the assembled KN400 genome sequence. All of these differences occur in a 30 bp region of the genome that is covered by just one 454 read. This means that the accuracy of this short region is a direct reflection of the quality of the single overlapping read. For this work, we utilized first generation short read technologies, and read quality and quantity have dramatically improved thereafter. Such low coverage regions are rare even in our assembly, and with contemporary data output they would likely be nonexistent. That is, investigators using our approach with the improved read data would almost certainly not have such assembly errors because 1) there would likely be no such regions since data output is so high and 2) the accuracy of individual reads is so much higher. It is worth pointing out that any approach based on short reads would be limited by the single 454 reads spanning this region, but what sets our approach apart is that we were able to actually place this single read in the context of assembling a circularly-closed bacterial genome.

### KN400 is the first microbial genome that has been completely assembled *de novo* using only next-generation sequencing technologies

**Figure 3:**
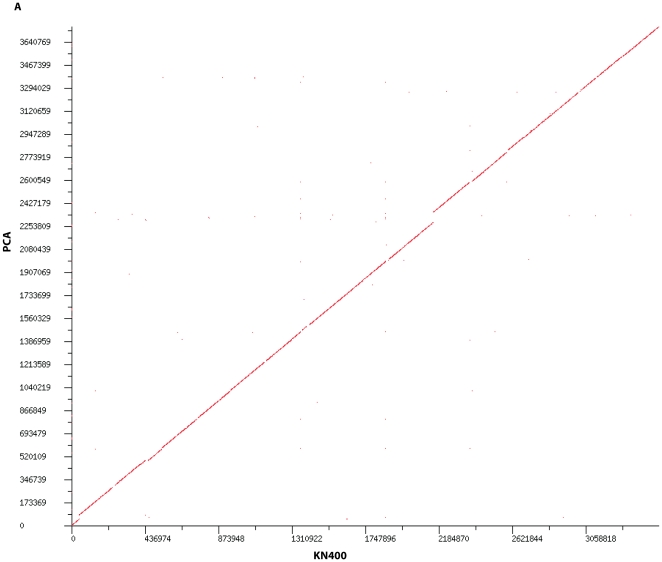
Genome-level comparison of KN400 and PCA. Shown in this figure is a dot-plot of the genome-wide alignment of KN400 and PCA. Along the X-Axis is the KN400 genome and the PCA genome is shown along the Y axis.

We predicted 3356 ORFs and 54 RNAs in the KN400 genome using the RAST pipeline [13] and manual curation. We found the completed KN400 genome to be collinear over its entire length with no major rearrangements (Figure 3)–and approximately 97% identical at the sequence level to *Geobacter sulfurreducens* PCA [14]. Because the genomes were so similar, we evaluated the correctness of our *de novo* assembly by assessing the commonalities and the differences between PCA and KN400 genomes. We adopted a comparative genomics approach at the ORF level to assess the differences and similarities between KN400 and PCA (Figure 2B).

#### Meta-Assembly approach facilitates accurate prediction of genomic regions unique to PCA

**Figure 4:**
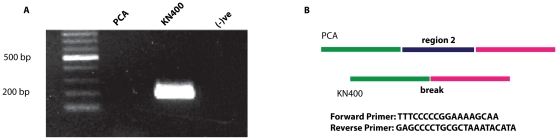
Gel picture confirming the 79 kb deletion (Region2) in KN400. PCR was performed with primer sets in order to amplify over the break (shown in panel B). The expected product size is 207bp. Panel A shows that we can amplify over the break only in KN400 and not in PCA, confirming the deletion of region 2 in KN400.

We identified three primary genomic regions that were specific to the PCA strain and had no correspondence with the KN400 genome: a 32kb region between GSU0039 and GSU0064 (in KN400) (Region 1), a 78kb region between GSU2105 and GSU2183 (Region 2), and a 16 kb region between GSU2588 and GSU2601 (Region 3) (Table S3). The largest region, GSU2105-GSU2183, had 79 genes in PCA between the orthologs to ORFs KN400_2161 and KN400_2163. Forty-six of these genes were hypothetical, and 11 were transposases or other integrative genetic elements. The remaining genes were predicted to encode several sensors and regulators and a DNA-binding repair protein (region 2 in Table S3). The deletion of this region was confirmed by performing a PCR over the break (Figure 4). The second largest region, GSU0039-GSU0064, had 26 genes in PCA between the orthologs to KN400_0039 and KN400_0040. Fifteen of these were hypothetical and 4 were transposases (region 1 in Table S3). In addition, there were 3 CRISPR-associated proteins. The third region, GSU2588-GSU2601, had 11 genes in PCA between the orthologs to KN400_2568 and KN400_2571. Four of these were transposases and seven hypothetical proteins (region 3 in Table S3). The abundance of transposons in these PCA-strain-specific regions suggests that they resulted from the activity of mobile genetic elements within the *Geobacter sulfurreducens* genome since the time the two strains diverged. None of these regions encoded any protein predicted to be required for growth in the PCA strain [15].

#### Genomic Regions unique to KN400 relative to PCA provides insights into possible evolutionary paths for KN400

Of the 3356 ORFs predicted in KN400, we found that 3088 (92%) had complete reciprocal orthologs with the same relative ordering in PCA. Conservation of the orthologs between the two genomes was quantified by the ratio of the bit scores from a KN400-PCA to a KN400-KN400 BLAST alignment. The orthologs had an average bit score ratio of 93.0% (Table S4).

We compared the remaining 268 proteins in KN400 that did not have reciprocal orthology in PCA to all proteins in the NCBI RefSeq database [16] to determine the organism which encoded the protein with the highest sequence similarity (Table S4). Fifty-six proteins had no significant match in the database, indicating they were specific to the KN400 genome, and were annotated as hypothetical proteins (Table S4). Thirty-six proteins were most similar to PCA, and 90 were most similar to other *Geobacteraceae*, indicating that they were vertically inherited (Table S4). This left 86 proteins that were found in the KN400 but were most similar to a non-*Geobacteraceae* species (Table S4). These 86 genes were found primarily in 12 small regions in KN400 that aligned poorly with the PCA genome (Table S4). These regions ranged in size from 3.3 to 21.2kb, and seven of them included genes for at least one transposase or integrase. In particular, copies of the two-subunit transposase ISGsu7 were associated with strain-specific islands in both PCA and KN400 (Tables S3 and S4). This supports the idea that these regions may also have been produced by the activity of mobile genetic elements since the two strains diverged.

*C*-type cytochromes play a key role in the transfer of electrons from central metabolism to external electron acceptors like Fe(III) and electrodes [17]. Comparative analysis of KN400 and PCA showed that several genes encoding cytochromes contain single nucleotide polymorphisms between the strains, including the gene for the outer-membrane cytochrome OmcS (GSU2504), which has been shown to be required for electron transfer to insoluble Fe(III) [18]. In addition, analysis of the genes specific to the KN400 strain also shows that there are at least three transport proteins that are not found in the PCA strain (Table S4). Further analysis of the link between these types of genetic differences and the phenotype of the KN400 strain during electricity production is underway (Butler J.E. et al, in prep).

### Genomic characteristics of KN400 are typical of microbial genomes at large

**Figure 5:**
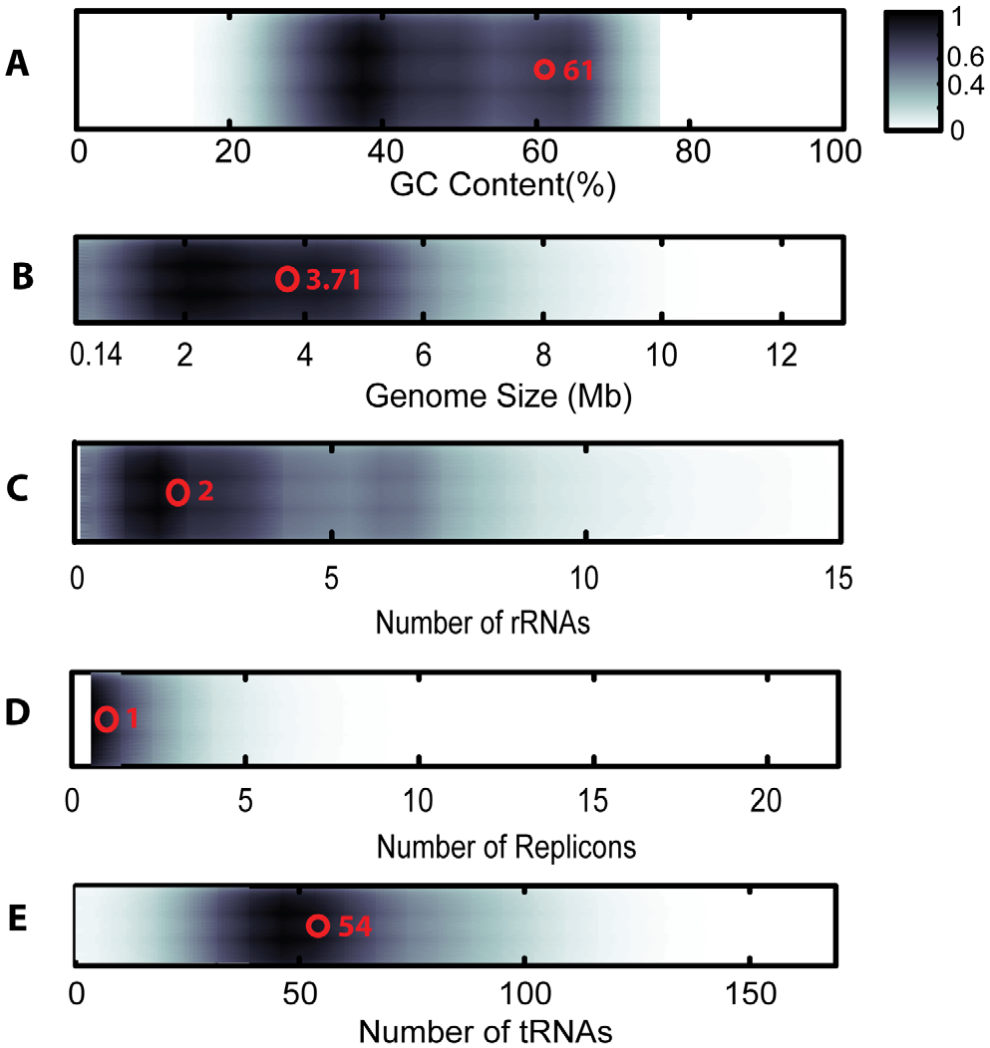
Density of five different genomic properties in the space of microbial genomes. A) GC Content B) Genome Size C)Number of rRNAs D) Number of Replicons E) Number of tRNAs. Shown in red circle, is the value of KN400's genomic property.

To assess whether KN400 was a particularly fortuitous choice for attempting a *de novo* assembly using only short reads, we assessed its genome complexity by performing a comparative analysis using five different genomic properties that have been characterized using 895 microbial genomes. We obtained data relating to the GC content, genome size, number of replicons, number of rRNAs and number of tRNAs for a sample of 895 microbial genomes from the Genome Atlas Database (http://www.cbs.dtu.dk/services/GenomeAtlas-3.0/). We computed the percentile ranks for KN400 genome to evaluate its relative complexity in the space of all microbial genomes across these five dimensions (Figure 5). Based on size and GC content, the KN400 genome has percentile ranks of 53 (3.71 Mb) and 75 (61% G+C). Furthermore, about 60% of the microbial genomes consisted of a single replicon – as is the case with KN400. KN400 has two ribosomal RNA operons, which is the most frequent number of rRNA operons among the 895 microbial genomes. Apart from large repeats like the rRNA operons, local inverted repeats like transposases also characterize genomic complexity and thus have an effect on the assembly process. To evaluate the transposase content of KN400, we relied on a recent survey [19] of the transposases present in all available microbial genomic and metagenomic databases. This survey found that the average genome contains 11 transposases per 1kb. Based on this result, a genome the size of KN400 would be expected to contain 38 transposases. Our analysis indicated that KN400 contains 30 transposases. Based on these comparisons, we concluded that KN400 is a typical microbial genome and not an outlying “simple” genome.

## Discussion

*De novo* assembly of complete microbial genomes using new DNA sequencing technologies and without the aid of Sanger sequencing has been an unsolved challenge. We have developed a bi-level integrative approach, the Meta-Assembly, that answers this challenge.

Our Meta-Assembly strategy is composed of four key phases. In phase one we integrated Illumina and 454 reads at the very beginning of our assembly process to generate hybrid contigs, instead of using Illumina reads only for error correction of an assembly generated from just 454 reads [7]. This early integration step was very important for reducing the number of degenerate nucleotide positions (Tables 1 and 3) and thus for the overall quality of the assembly. Incorporating the Illumina reads early in the assembly process significantly reduced the number of degenerate nucleotides in the assembly (∼41000 N's) compared to when they are used for just error correction of the assembly generated by Newbler (∼90,000 N's). In addition, we used EULER-SR [1] instead of VCAKE [3] as the short read assembler–in distinction from an earlier report [6]. The fact that de-Bruijn graph based algorithms like EULER-SR [1] and Velvet [2] outperform VCAKE [3] has been documented in an earlier study [20], and we found the same trend with our data as well. Since assembly of Illumina reads is the first step of the hybrid assembly phase, the quality of the initial assembly has the greatest impact on the outcome of the entire process. Moreover, EULER-SR [1] is also capable of performing a *de novo* assembly with a mixture of Illumina and 454 reads, but its performance does not degrade with increasing read length ([20]). This proved to be a significant advantage, for we were able to exploit the complementary nature of EULER-SR [1] and Newbler to develop the Scaffold Bridging and Finishing Phase–enabling us to resolve all of the degenerate nucleotides.

**Table 3:** Comparison of Meta-Assembly to other assembly programs.

Assembler	Number of Contigs/Scaffolds	N50(kb)	Degenerate Positions	Assembly Length(Mb)
Meta-Assembly	1	3714.2	0	3.71
EULER-SR	150	58	0	3.70
Velvet	329	45	486532	3.89
Newbler alone	5	3184.3	90449	3.72

In the second phase, we maximized the complementary information provided by different assembly algorithms. This component of our strategy is a key distinguishing aspect of our approach. Although Newbler alone was able to assemble the reads into five scaffolds, the resulting assembly had a considerable number of degenerate positions which could not be resolved just from an error correction step using Illumina reads (Table 3). Similarly, while EULER-SR [1] and Velvet [2] both generated high quality contigs, they do not perform as well as Newbler with respect to leveraging the paired-end information in the 454 reads. Our results clearly show that integrating more than one assembly algorithm is very important for enhancing the quality of the assembly.

In the third phase, the simple PCR-based search strategy allowed us to quickly order and orient the scaffolds into a circular genome. This is another unique aspect of our approach in that we address the problem of relative orientation of the scaffolds as well as their ordering with just a few PCRs. While we use the PCRs to order the scaffolds into a circular genome, we did not fill any gaps as no sequence information is obtained from the PCRs. We note that as technology improvements allow paired-end sequencing reads with longer inserts, the necessity of this PCR step will decrease.

In the fourth and final phase, we aligned Illumina reads against the ordered scaffold to account for indels and errors induced during the scaffold finishing phase.

To our knowledge this is the first reported *de novo* assembly of a complete genome using next generation sequencing technologies. Furthermore, our comprehensive comparative analysis of genomic characteristics of 895 microbial genomes reveals that KN400 is a characteristic microbial genome and is not an outlier in the space of all microbial genomes. We view our result as the demonstration of general a strategy for assembling genomes, wherein multiple data types are integrated at specific steps in the process to maximize the potential of their complementary nature and wherein multiple assembly programs are utilized such that deficiencies in one algorithmic approach are compensated by the strengths of another algorithmic approach. As new sequencing technologies and new assembly programs become available, they can be readily incorporated in this framework. Genome assembly will remain challenging for the foreseeable future, and we view the idea of such a readily extensible meta approach as one of the most promising ways to meet this challenge.

## Materials and Methods

### Data

We utilized an electrode isolated strain of a rare variant of *Geobacter sulfurreducens* KN400*t*hat exhibits enhanced current production relative to *Geobacter sulfurreducens* PCA [10]. For the purpose of a *de novo* assembly, we carried out both Illumina sequencing and 454 Life Sciences pyrosequencing (shotgun and paired end) for KN400. The details of the sequence data used are shown in the Table S1. The paired end reads had an average insert size of 3020 bp and the distribution of the fragment size is given in Supplementary Figure S1.

### Meta-Assembly

#### Hybrid Assembly Phase

We assembled Illumina GA1 reads using the de-Bruijn graph based short-read assembler EULER-SR [1] with the vertex size parameter set to 25. Prior to assembling the short-reads, we filtered them for obvious failure modes using the filterIlluminaReads script as part of the EULER-SR package [1]. We took all of the 454 reads as singletons (i.e. neglecting the paired end information) and assembled them using Roche's assembler (Newbler) with default parameters (minimum overlap length 40, minimum overlap identity 90%). In order to integrate the Illumina and 454 technologies, we combined the contigs generated from the EULER-SR [1] and Newbler assemblies using the incremental assembly option in Newbler to generate a set of hybrid contigs. At this stage, we leveraged the mate-pair information provided by the 454 reads in order to build scaffolds from these hybrid contigs.

#### Scaffold Bridging and Finishing Phase

In order to maximize the complementary nature of the assembly algorithms, we developed a meta-approach that reconciles the assemblies produced by either EULER-SR [1] or Velvet [2] and Newbler. The *de novo* scaffolds generated from phase A are largely due to Newbler and contain a lot of degenerate nucleotides. We created a second set of hybrid contigs from both the Illumina and 454 reads using EULER-SR with a vertex-size of 25. Using the NUCMER alignment tool of MUMMER package [21], we aligned these hybrid EULER-SR [1] contigs against the *de novo* scaffolds with the break length parameter of 10,000. We further implemented a custom program (Supplementary Figure S2) which accounted for the following three scenarios, in order to link up the *de novo* scaffolds and resolve the degenerate nucleotides.

- If any of these contigs aligned in such a way that they were bridging any two of the *de novo* scaffolds, those scaffolds were linked up (Figure S2A).
- In the event that these contigs overlapped with the flanking regions of degenerate nucleotides (N's), we replaced the N's with the corresponding region of the contig.(Figure S2B)
- If the regions flanking the degenerate positions aligned to contiguous regions in the contig set, we removed that stretch of degenerate nucleotides (Figure S2C).

We further augmented this custom program with an alignment of Illumina reads against these scaffolds in order to account for the indel errors due to 454 sequencing. We used the MosaikAligner in the “all” alignment mode with a hash size of 13 and 20 bp as alignment candidate threshold, allowing a maximum of 4 mismatches.

#### Scaffold Ordering Phase

We considered the KN400 genome as a signed circular permutation of the finished scaffolds and adopted a PCR based scaffold ordering approach in order to orient them into a circular genome. We designed a search strategy comprised of nine PCRs in order to determine the correct orientation. For performing the PCRs, total *G. sulfurreducens* (KN400) genomic DNA was prepared using the MasterPure Complete DNA Purification kit (Epicentre Biotechnologies, Madison, WI) according to manufacturer's directions. *Taq* DNA polymerase (QIAGEN Inc., Valencia, CA) was used for all PCR amplifications. The sequence and the location details of the primer sets used for the PCRs are provided in Table S2A, while the combinations of the primer pairs for each PCR and the expected amplicon size are given in Table S2B.

#### Genome Finishing Phase

In order to account for any possible indel errors due to the 454 sequencing as well as our Meta-Assembly approach, we re-aligned all of the Illumina reads to the ordered scaffold using MosaikAligner (Stromberg and Marth in preparation) with the same parameters as described in Phase B of the Meta-Assembly approach.

### Validation by Sanger Sequencing

We validated our assembly approach and computed error rates by performing Sanger sequencing of about 1% of the KN400 genome. The regions that were selected for sequencing along with the details of the primers used for PCR amplification are shown in Table S5.

### Gene Prediction and Comparative Analysis

At each step of the finishing process, we predicted open reading frames (ORFs) in the KN400 draft genome using the Rapid Annotation by Subsystem Technology (RAST) pipeline [13] in order to check if the standard properties like gene density were characteristic of a bacterial genome. We performed a whole genome alignment of KN400 and PCA using Mauve (Darling et al., 2004) with a seed-weight of 15, a minimum island size of 50, and the Muscle 3.6 algorithm in order to check for completeness of all the homologous gene sequence. We further confirmed the absence of any genes with respect to PCA by aligning the Illumina reads to the corresponding region.

#### Comparative Genomics Analysis

We obtained the PCA genome sequence, translated ORFs, and functional annotation from NCBI (RefSeq ID NC_002939) and the RefSeq database [16] and performed an alignment using BLAST [11]. We extracted the Identifiers, functional annotations, and organism names for the closest matches for all ORFs. We calculated bit score ratios as the bit score of the best match in a KN400-PCA or KN400-RefSeq BLASTp or tBLASTn comparison adjusted by the bit score of the KN400 protein aligned to itself or its own genome. Orthologs were defined as reciprocal best matches in whole-genome KN400-PCA BLASTp comparisons [22]. We also performed a bidirectional Smith-Waterman alignment of the ORFs of KN40 and PCA using the *ssearch35* program of the FASTAv3.5 package [23].

## Supporting Information

Figure S1 Distribution of fragment sizes from 454 paired end sequencing data.
(0.44 MB TIF)

Figure S2 Custom program used in the Scaffold Bridging and Finishing phase.
(0.56 MB TIF)

Table S1 Next generation sequencing data used for *de novo* assembly of KN400.
(0.02 MB XLS)

Table S2 A: Sequence and location of primers. (Lower case denotes reverse complement.) B: Primer pairs and product analysis of each PCR.
(0.03 MB XLS)

Table S3 Genomic regions absent in KN400 but present in PCA.
(0.04 MB XLS)

Table S4 All predicted KN400 genes with annotation and comparative analysis.
(0.95 MB XLS)

Table S5 Selected regions for Sanger sequencing, coordinates and primer details.
(0.05 MB XLS)
