# Supplementary figures and images for: De Novo Assembly of the Complete Genome of an Enhanced Electricity-Producing Variant of Geobacter sulfurreducens Using Only Short Reads
Source: PLoS One. 2010 Jun 8;5(6):e10922. doi: 10.1371/journal.pone.0010922 (PMC2882325; doi:10.1371/journal.pone.0010922)

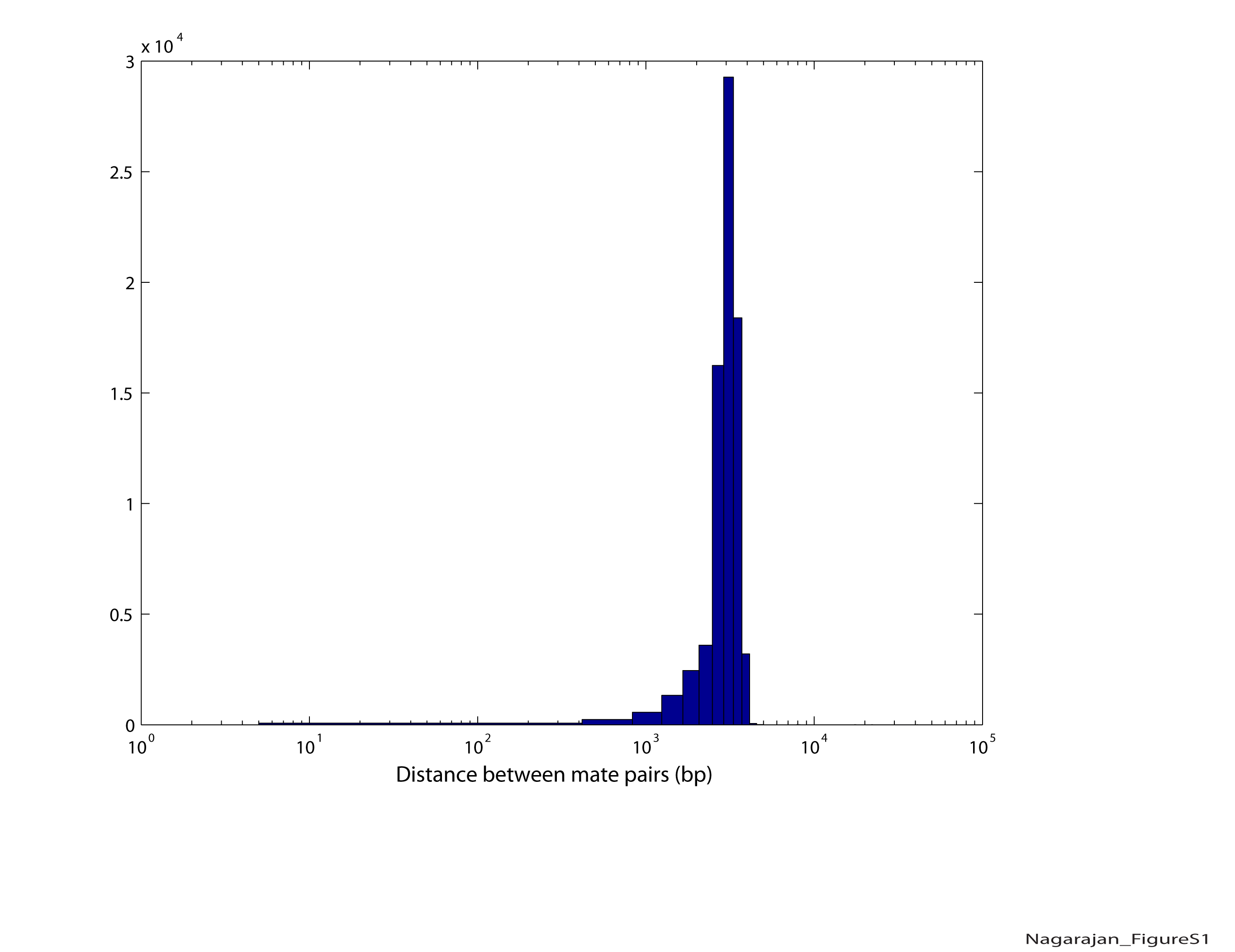

Supplement: Figure S1 — Distribution of fragment sizes from 454 paired end sequencing data. (0.44 MB TIF) [file pone.0010922.s001.tif]

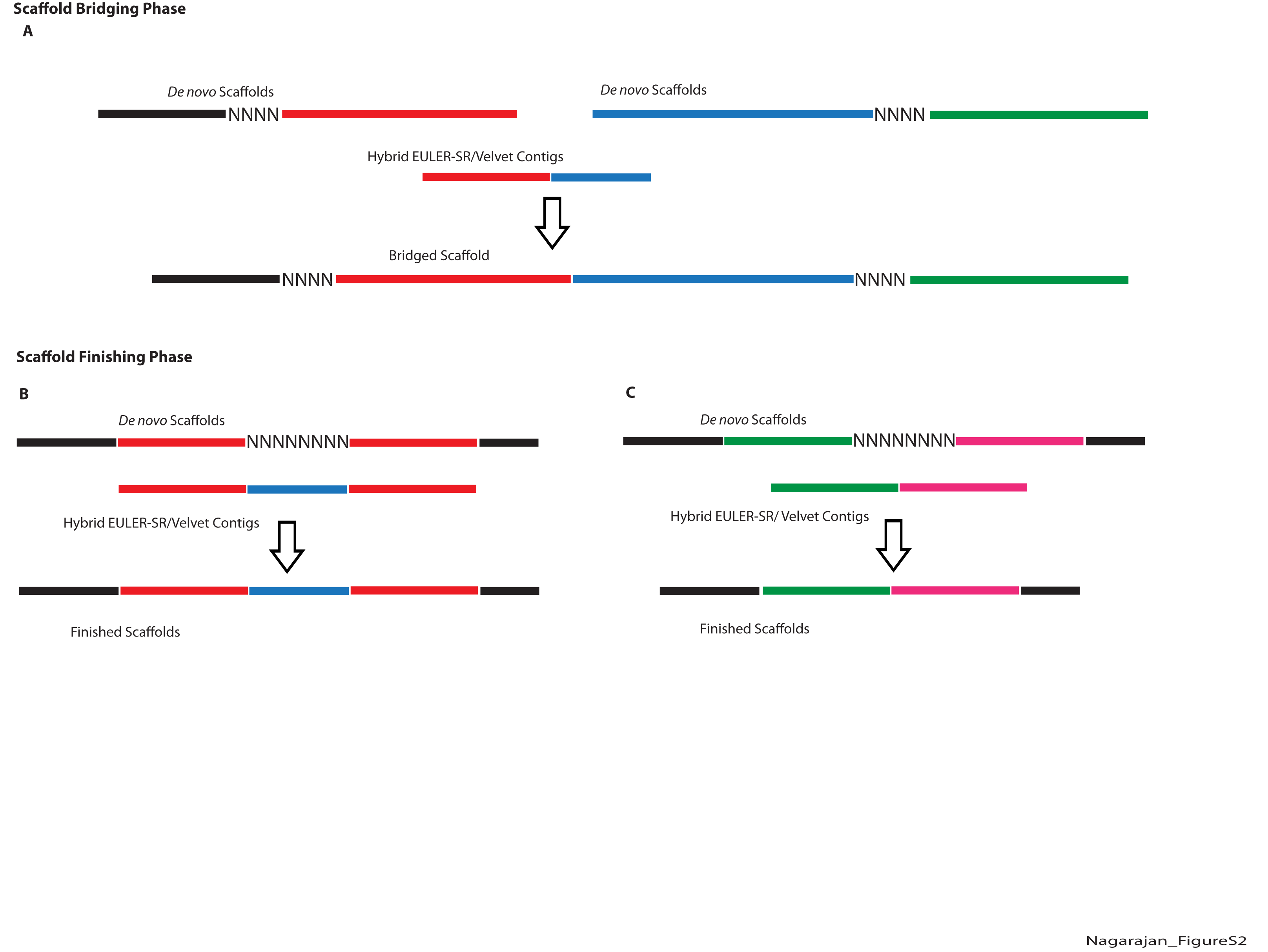

Supplement: Figure S2 — Custom program used in the Scaffold Bridging and Finishing phase. (0.56 MB TIF) [file pone.0010922.s002.tif]
